# Emerging roles for UDP-glucuronosyltransferases in drug resistance and cancer progression

**DOI:** 10.1038/s41416-019-0722-0

**Published:** 2020-02-12

**Authors:** Eric P. Allain, Michèle Rouleau, Eric Lévesque, Chantal Guillemette

**Affiliations:** 1 Pharmacogenomics Laboratory, Centre Hospitalier Universitaire (CHU) de Québec Research Center—Laval University, Québec, QC Canada; 2 Faculty of Pharmacy, Laval University, Québec, QC Canada; 3 Faculty of Medicine, Laval University, Québec, QC Canada

**Keywords:** Chronic lymphocytic leukaemia, Cancer therapy

## Abstract

The best-known role of UDP-glucuronosyltransferase enzymes (UGTs) in cancer is the metabolic inactivation of drug therapies. By conjugating glucuronic acid to lipophilic drugs, UGTs impair the biological activity and enhance the water solubility of these agents, driving their elimination. Multiple clinical observations support an expanding role for UGTs as modulators of the drug response and in mediating drug resistance in numerous cancer types. However, accumulating evidence also suggests an influence of the UGT pathway on cancer progression. Dysregulation of the expression and activity of UGTs has been associated with the progression of several cancers, arguing for UGTs as possible mediators of oncogenic pathways and/or disease accelerators in a drug-naive context. The consequences of altered UGT activity on tumour biology are incompletely understood. They might be associated with perturbed levels of bioactive endogenous metabolites such as steroids and bioactive lipids that are inactivated by UGTs or through non-enzymatic mechanisms, thereby eliciting oncogenic signalling cascades. This review highlights the evidence supporting dual roles for the UGT pathway, affecting cancer progression and drug resistance. Pharmacogenomic testing of UGT profiles in patients and the development of therapeutic options that impair UGT actions could provide useful prognostic and predictive biomarkers and enhance the efficacy of anti-cancer drugs.

## Background

**Fig. 1:**
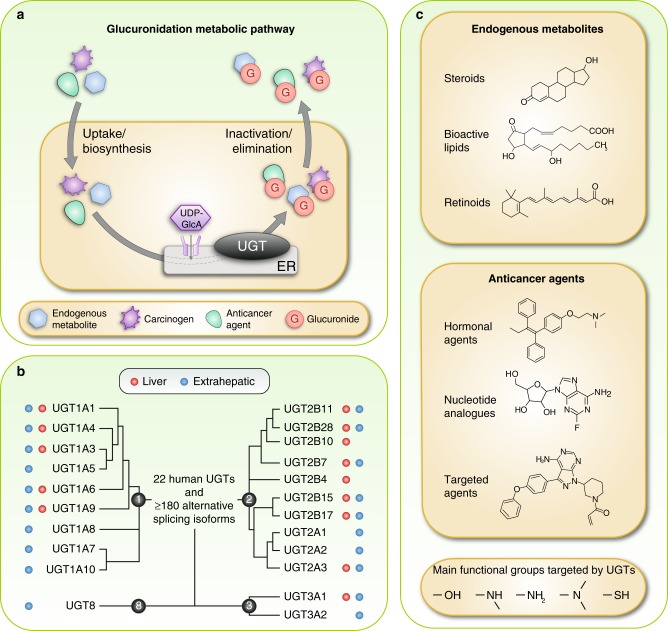
Schematic overview of the glucuronidation reaction catalysed by UGT enzymes. **a** Endogenous metabolites, carcinogens and drugs are conjugated to glucuronic acid (GlcA) taken from the preferential co-substrate UDP-GlcA by membrane-bound UDP-glucuronosyltransferase (UGT) enzymes. The glucuronidation reaction generally takes place in the lumen of the endoplasmic reticulum (ER), and requires active transport of cytosolic UDP-GlcA to the ER. **b** Four *UGT* families (*UGT1, UGT2, UGT3* and *UGT8*) encode the 22 enzymes and alternative isoforms (≥180) that regulate the glucuronidation pathway in humans. UGTs are found in most organs, generally anchored to the luminal side of the ER. Some UGTs may also reside in the perinuclear membrane (not illustrated). The liver expresses the widest array of UGTs. Enzymes of the UGT3 and UGT8 families use the co-substrates UDP-glucose, UDP-xylose, UDP-galactose and UDP-N-acetylglucosamine rather than UDP-GlcA to catalyse the glycosylation of endogenous metabolites (see Box 1). **c** Examples of endogenous metabolites and anti-cancer agents targeted by UGTs. Hydroxyl, amine, and sulfhydryl are the main functional groups targeted by UGT enzymes. Each UGT is specialised in the conjugation of a specific set of substrates.

One mechanism by which cancer cells promote their own growth and survival is by altering the biotransformation of small molecules or metabolites. Conjugation reactions that are catalysed by UDP-glucuronosyltransferase enzymes (UGTs) constitute one such means of biotransformation. In humans, the UGT pathway is mediated by 22 enzymes that catalyse the covalent addition of sugars from nucleotide UDP-sugar donors to hydroxyl, carboxyl or amino groups of a diversity of dissimilar endogenous metabolites and toxic exogenous chemicals (Fig. 1).^1^ Because of their significant role in drug metabolism, the UGT1 and UGT2 subfamily members are the most studied. In the vast majority of cases, conjugation of lipophilic substrates by UGT1 and UGT2, using highly polar glucuronic acid (GlcA) for the formation of hydrophilic glucuronides—a reaction known as glucuronidation—abolishes biological activity, enhances solubility and facilitates elimination from the body through bile and urine (Fig. 1a) (Box 1).

**Fig. 2:**
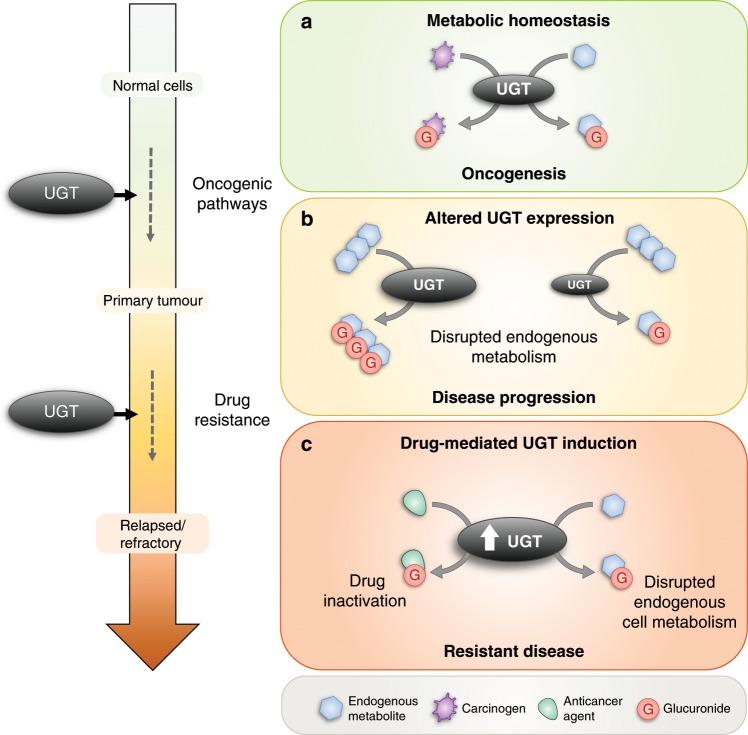
UGTs as mediators of oncogenic pathways and drug response. **a** UDP-glucuronosyltransferases (UGTs) regulate metabolic homoeostasis through the inactivation of endogenous metabolites (such as steroid hormones) and xenobiotics (such as carcinogens) by their conjugation with glucuronic acid (GlcA, G). **b** The modulation of UGT expression and activity observed in normal and cancer cells influences the bioactivity of metabolites, including some with oncogenic potential, thereby promoting tumour development and progression. Endogenous metabolites such as steroid hormones induce or repress the expression of specific UGTs. **c** Therapeutic drugs generally induce UGT expression, promote drug inactivation and resistance to treatment and further perturb endogenous metabolites.

UGT enzymes are highly expressed in metabolic tissues such as the liver, intestine and kidney, consistent with their role in facilitating the elimination of certain metabolites, but the expression of most UGT subfamily members also extends to many other organs and blood cells (Fig. 1b). The tissue-specific expression of UGTs is highly regulated by multiple signalling pathways and transcription factors, and might be linked to the substrate metabolites they conjugate.^2,3^ The substrate molecules can be from endogenous or exogenous sources (Figs. 1c, 2a).^4^ For example, under homoeostatic conditions, glucuronidation is used by prostate epithelial cells to mediate the inactivation and elimination of androgens, thereby controlling their potency and availability. Additionally, UGTs, often acting in concert with other drug-metabolising enzymes and transporters, can participate in the inactivation of xenobiotics comprising environmental and dietary toxins and pharmacological compounds including anti-cancer drugs. Significantly, drug inactivation by UGTs is emerging as an important mechanism of drug resistance in cancer (Fig. 2c).

In testament to the importance of UGTs in cancer, a number of studies have established links between genetic variability in UGT enzymes and the risk of developing cancer in many different tissues, supporting roles for UGTs in the detoxification of exogenous carcinogens and inactivation of endogenous tumour-promoting molecules. These links have been extensively reviewed and will not be discussed further.^5–7^ Less-well considered are the associations between UGTs and cancer progression, and UGTs and resistance to therapy, either primary or acquired, which we review herein.

Box 1 Human UDP-glycosyltransferases. The human glycosyltransferase enzymes represent a superfamily of proteins that catalyse the addition of sugar residues from a nucleotide-sugar donor (the co-substrate) to a functional group (usually a hydroxyl, carboxyl, amine or sulfhydryl group) of small lipophilic chemicals (generally below 1500 Da). The best characterised conjugation glycosyltransferases are UDP-glucuronosyltransferases (UGTs) of the subfamily UGT1 and UGT2. UDP-GlcA is the widely preferred co-substrate of UGT1 and UGT2 enzymes, and hence, the catalysed conjugation reaction is referred to as glucuronidation. UGTs are membrane-bound enzymes. They predominantly localise in the endoplasmic reticulum (ER), where the glucuronidation reaction takes place on the luminal side of the ER. Some UGTs may also reside in the perinuclear membrane. UGTs share a structural organisation consisting of a N-terminal co-substrate binding domain, a C-terminal substrate binding domain linked to a transmembrane region and a positively charged lysine-rich tail facing the cytosol.

The glucuronidation reaction occurs by a second-order nucleophilic substitution. The nucleophilic attack of the co-substrate UDP-GlcA by the polar functional group of the substrate enables the transfer of the GlcA from the co-substrate to the substrate. The end products of the reactions are the glucuronidated metabolite and UDP. The main glucuronides formed in humans are *O*-linked and *N*-linked. Glucuronidated metabolites have their water solubility enhanced relative to the parent molecule, facilitating their elimination in the bile and urine. For the most part, glucuronidated metabolites have reduced or abolished biological activity relative to the parental compounds. One notable exception is the glucuronidation of morphine by the enzyme UGT2B7, which produces morphine-6-glucuronide with enhanced analgesic activity relative to morphine. Hence, UGTs have essential detoxification and clearance functions by regulating the metabolism, bioactivity and bioavailability of pharmacological, dietary and environmental compounds, as well as of endogenous molecular substrates such as bile acids, signalling lipids, vitamins and steroid hormones. The 19 UGT1 and UGT2 enzymes modulate the pharmacological efficacy of about 55% of the most prescribed drugs.^1^

The three members of the other two human subfamilies, UGT3A1, UGT3A2 and UGT8 are less well characterised. Latest studies have revealed that these enzymes, named on the basis of amino acid sequence similarities, do not use UDP-GlcA as a co-substrate. UDP-N-acetylglucosamine is the preferred UGT3A1 co-substrate, whereas UDP-glucose and UDP-xylose are the main co-substrates of UGT3A2.^8,9^ UGT8 is rather known as a UDP-galactosyltransferase, mostly expressed in the brain but also detected in other organs, and was first described as a ceramide-glycosyltransferase.^10^

## UGTs as mediators of drug resistance

**Fig. 3:**
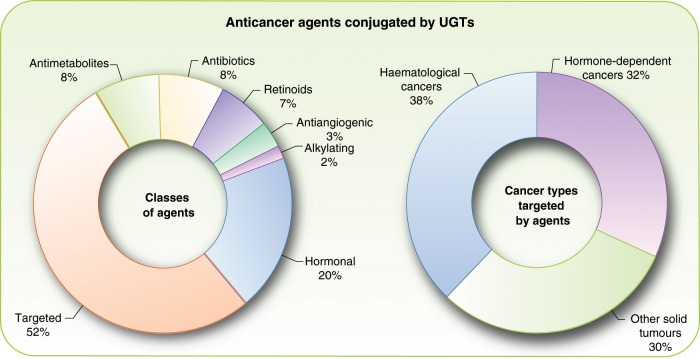
Cancer therapeutic drugs of all classes and targeting all types of cancers are regulated by the glucuronidation pathway. A non-exhaustive list of over 70 therapeutic agents inactivated by UGTs is provided in Supplementary Table S1.

The contribution of glucuronidation to the response and resistance to cancer therapies was first recognised nearly 30 years ago, when a link between the high efflux of anthracycline (daunorubicin) as a glucuronide conjugate from a leukaemic cell line and cell resistance to daunorubicin cytotoxicity was demonstrated.^11^ The results from this study, and two other early cell-based studies on the active metabolite of irinotecan, SN-38, and on mycophenolic acid, hinted that intrinsic UGT expression and activity could significantly influence drug sensitivity and efficacy.^12,13^ Since this pioneering work, the list of anti-cancer drugs shown to undergo glucuronidation has rapidly grown, and includes the majority of currently used antineoplastic drugs of all classes and many others in development, targeting both haematological cancers and solid tumours (Fig. 3; Supplementary Table S1).^1^ For several of these therapeutic agents, including irinotecan, sorafenib, raloxifen and tamoxifen, glucuronidation by UGTs might considerably reduce drug activity and exposure (Supplementary Table S1).^14–18^

### Preclinical evidence of UGT-associated primary drug resistance

Much of the early evidence for the influence of UGTs on drug responses was derived from cell-based assays. For example, SN-38 is glucuronidated by several UGT1As,^19^ and colon, lung and breast cancer cells that show resistance to SN-38 have high expression levels of UGT1A and high levels of extracellular SN-38−glucuronide, consistent with low intracellular levels, and consequently, reduced exposure to the active drug.^13,20–22^ In parallel, pharmacogenetic studies of colorectal cancer patients established associations between germline variants of *UGT1* and clinical outcomes, and supported the role of the UGT1A enzyme pathway in mediating the response to SN-38.^1,23^ The best example pertains to the clinically actionable marker UGT1A1*28, which is associated with reduced UGT1A1 activity and irinotecan-induced severe neutropenia, leading to recommendations for dosing adjustment.^18,23^ This observation warrants further studies to clarify the influence of UGT expression levels and activity on clinical outcomes for irinotecan-based treatments, beyond the impact of germline polymorphic variants.

Similarly, the efficacy of novel therapeutic strategies has also been reported to be modulated by UGT expression. Ganetespib (STA-9090) and luminespib (AUY922) are promising next-generation anti-cancer drugs that target the heat-shock protein HSP90, an ATP-dependent molecular chaperone key to the folding of a large set of proteins including several oncogenic drivers.^24^ The main HSP90 inhibitors compete for the binding of HSP90 to ATP, and promote the proteasomal degradation of unfolded client proteins. The clinical effectiveness of ganetespib and luminespib is thus anticipated for multiple cancers, and they are currently being tested in several ongoing Phase 2/3 clinical trials, either alone or in combination therapies.^25^ However, in a gene expression analysis, elevated levels of UGT1A were among the most notable differences observed between drug-resistant and drug-sensitive colorectal and bladder cancer-cell lines exposed to these HSP90 inhibitors.^26,27^ In addition, overexpression of UGT1A10 (which is abundantly expressed within the gastrointestinal tract) in a sensitive colorectal cancer-cell line rendered it resistant to the resorcinol-based inhibitors ganetespib and luminespib, whereas the knockdown of UGT1A in resistant colorectal and bladder cancer-cell lines increased drug sensitivity.^26,27^ By contrast, the same colorectal and bladder cancer-cell lines were all sensitive to other classes of HSP90 inhibitors such as ansamycin-like drugs, which are not substrates of UGTs.^26,27^ The resistance mechanism against resorcinol-based inhibitors was thus convincingly associated with their glucuronidation in resistant colon and bladder cancer-cell lines, and the glucuronide derivatives they produced correlated with UGT1A expression.^26–28^ Clinical investigations are needed to address this resistance pathway in colorectal and bladder cancer patients. Although earlier reports suggested that colorectal and bladder tumours showed reduced UGT1A expression relative to normal tissues,^29–31^ other studies indicated that a significant subset of colorectal tumours display high levels of expression,^32,33^ similar to those observed in resistant cell lines, suggesting that such patients would not benefit from resorcinol-based HSP90 inhibitors.^26,27^ Based on the results of a sensitivity screen comparing low and high UGT1A-expressing cancer-cell models, UGT1As might be important determinants of the response to several other novel antineoplastic compounds—those that are Food and Drug Administration (FDA)-approved or currently in clinical trials—including methotrexate, the farnesyltransferase inhibitor tipifarnib and the vascular endothelial growth factor (VEGF) inhibitor pazopanib.^26,34^

### Clinical evidence of UGT-associated primary drug resistance

The expression of UGT1A has been associated with the response to the epidermal growth factor receptor (EGFR) inhibitor erlotinib in patients with non-small-cell lung cancer (NSCLC) or head and neck cancer.^35,36^ Although studies were conducted by using a limited number of patients, the tumour levels of *UGT1A* mRNA were threefold higher in non-responding patients with head and neck cancer, and those of *UGT1A6* mRNA were eightfold higher in non-responding patients with NSCLC prior to treatment. Consistent with these correlative data, glucuronide conjugation of erlotinib oxidative metabolites is a documented route of its inactivation and elimination,^37^ suggesting that elevated levels of *UGT* reduce sensitivity to erlotinib. Resistance might also be mediated by an inhibitory effect of erlotinib on UGT1A1-mediated glucuronidation within the tumour cells.^38^ For example, several tyrosine kinase inhibitors have been shown to inhibit UGTs, leading to drug–drug interactions that may influence response to other anti-cancer drugs by altering the elimination of co-administered drugs.^39^ The potent inhibition of UGTs by tyrosine kinase inhibitors could also potentially have a significant clinical effect on the metabolism of endogenous oncogenic substrates involved in cancer-cell progression. The drug–drug and drug–endobiotic interactions appear as two independent mechanisms that can operate concurrently to alter local drug response.

*UGT1A6* is also expressed at significantly higher levels in tumours from patients with advanced renal clear-cell carcinoma who do not respond to the programmed cell death 1 (PD-1)-blocking antibody nivolumab—288-fold higher expression than in responders, on average—along with a more modest, but significantly higher, expression of *UGT1A1* and *UGT1A3* (by 5- and 7.1-fold, respectively). This study was conducted in a small group of patients (four responders and eight non-responders), but the mRNA expression data are supported by immunohistochemical observations, with an average of 65% UGT1A6-positive cells in tumours from non-responders relative to 23% in tumours from responders.^40^ Expression was measured before treatment initiation, consistent with UGT1A6 being a predisposing resistance factor, as for patients with head and neck cancer or NSCLC. This finding is intriguing given that, in contrast to small molecules such as erlotinib that are conjugated by UGTs, nivolumab is an immunotherapeutic antibody against PD-1 and an unlikely substrate of UGTs that are not known to conjugate proteins. This raises the possibility that in some oncogenic contexts, UGT-mediated impairment of a drug response might not involve direct drug inactivation. Instead, the underlying mechanism could involve the regulation of bioactive metabolites and signalling molecules inherent to cancer cells, as discussed below. Nevertheless, these examples support the notion that the basal levels of UGT expression might have a crucial impact on the drug response in a drug-naive context, and highlight the value of UGT expression in primary tumours as predictive biomarkers.

## Inducible UGT expression and acquired drug resistance

The expression and activity of UGTs are tightly regulated, both in normal and tumour tissues. As well as the ability of epigenetic, transcriptional and post-translational factors to collectively shape the levels of UGTs in a given tissue,^1,41^ the expression of UGTs can also be influenced by endogenous metabolites and exogenous dietary, environmental and pharmaceutical factors. For instance, glucuronidation of ligands that activate transcription factors controlling UGT expression provides extensive feedback-regulated signalling and crosstalk, and allows fine regulation of xenobiotic and endobiotic signals. These influences could impact systemic exposure to drugs by altering UGT expression in drug-metabolising tissues and local exposure in tumour tissues. In the context of cancer treatments, acquired drug resistance driven by induced UGT expression is documented in several preclinical models as well as in patients with haematological malignancies.

### Preclinical evidence of acquired resistance mediated by induction of UGTs

The drug-induced expression UGT enzymes has been repeatedly observed after exposure of cancer-cell lines to anti-cancer drugs that are also UGT substrates. This observation raised the hypothesis that drug-induced UGT expression could result in higher drug inactivation and reduced drug sensitivity. This is the case for UGT2B7 induced by several anti-cancer agents in liver cell models, for UGT2B15 induced by tamoxifen and UGT2B17 induced by exemestane in breast cancer-cell models, as well as for UGT1A4 induced by fulvestrant in breast cancer and liver cell models.^42–46^

The notion that induction of UGT expression may lead to acquired drug resistance is further supported by the following studies. Breast cancer-cell models that were rendered resistant to methotrexate by prolonged exposure to the drug also displayed enhanced expression of several UGT1As—particularly UGT1A6—as well as enhanced glucuronidation activity.^47^ This effect was not observed in colorectal, pancreatic, leukaemia and osteosarcoma models made resistant to methotrexate in a similar manner,^47^ suggesting that UGT-dependent mechanisms of resistance might be specific to some cancer-cell-drug contexts and might be related to the specific UGTs that are expressed in the target organ. Inactivation of methotrexate by UGT1A enzymes has been proposed as a mechanism for resistance based on a high-throughput cell-based screen of multiple cancer drugs,^34^ which is consistent with resistance being related to drug glucuronidation by UGT1As.

Several UGT mRNAs are detected in normal skin, although the detoxification functions of UGTs in the skin have received little attention.^48^ UGT expression has been demonstrated in melanocytes derived from newborn foreskin tissues, with UGT2B isoforms (UGT2B7, UGT2B10 and UGT2B15) being expressed primarily in normal melanocytes as well as in a primary melanoma cell line.^48,49^ By contrast, UGT expression is undetected in three cell lines derived from untreated metastatic melanomas, which possibly indicates reduced UGT expression during melanoma progression.^49^ Treatment of these cell lines with any of the chemotherapeutic agents temozolomide, doxorubicin, epirubicin or vemurafenib does, however, induce expression of the same three UGTs.^49^ This induction is correlated with a reduced cytotoxicity of doxorubicin and epirubicin, but not of the non-substrate drugs temozolomide and vemurafenib. This result demonstrates the capacity of drug treatment to trigger the re-expression of UGTs and associated detoxification pathways to induce drug resistance. It also raises the important issue of cross-regulation of UGT expression by substrate and non-substrate drugs. The consequences of cross-regulation might be significant in chemotherapeutic contexts, given that one drug has the potential to induce the UGT pathway and create a novel form of multidrug resistance to other treatments inactivated by UGTs.^49^

### Clinical evidence of acquired resistance mediated by induced UGT expression in leukaemias

The significant contribution of UGTs to acquired drug resistance in cancer patients has emerged only in the last 6 years from studies of patients with chronic lymphocytic leukaemia (CLL) and acute myeloid leukaemia (AML) treated with nucleoside analogues, which are components of the first-line treatment of haematological malignancies.^50,51^ UGT2B17 is the main UGT expressed in leukaemic B cells from CLL patients, whereas all other UGTs are very low or undetected.^52^ The importance of UGT2B17 in the drug response was suggested by the results of a study of CLL patients treated with a fludarabine-based regimen.^50^ This study showed that non-responders to treatment could be distinguished from responders on the basis of the induced expression of *UGT2B17* observed as soon as few hours after treatment initiation. These observations indicate that the lack of response to the drug might be due to fludarabine inactivation by UGT2B17-dependent glucuronidation as well as the regulation of oncogenic pathways by UGT2B17 (Allain et al., unpublished data). These mechanisms might also apply to multiple other anti-leukaemic agents used to treat CLL, including venetoclax (Allain et al., unpublished data). In the cases of ibrutinib and idelalisib, which are not directly inactivated by UGT2B17 (Allain et al., unpublished data), the cellular response (or, rather, lack thereof) is suggestive of the regulation of oncogenic pathways by UGT2B17. However, it remains unknown whether this is mediated through enzymatic or non-enzymatic mechanisms (see below).

In another study, resistance of AML patients to treatment with ribavirin, another nucleoside analogue, was attributed to the induced accumulation of UGT1A enzymes and glucuronidation of ribavirin.^51^ The conjugation of another frequently used purine analogue, cytarabine, was also noted, suggesting that UGT-mediated resistance to this treatment might also arise as a consequence of UGT accumulation.^51^ Notably, however, transcription of *UGT1* in ribavirin- and cytarabine-resistant AML cells is reduced relative to untreated cells, and could not explain UGT accumulation; instead, levels of the UGT1A protein in these cells are enhanced by the inhibition of its proteasome-mediated degradation through a mechanism involving the sonic hedgehog transcription factor glioma-associated oncogene homologue 1 (GLI1) and potentially also the endoplasmic reticulum (ER) chaperone protein calreticulin.^34,51^

Collectively, these preclinical examples and clinical observations in colorectal cancer, renal cell carcinoma, head and neck cancers, NSCLC, AML and CLL all support the inherent and inducible expression of UGT, both at the mRNA and protein levels, as an important determinant of the drug response and drug resistance in cancer cells.

## UGTs and cancer progression

An emerging concept that is gaining support from several studies is the influence of UGTs on cancer progression by their ability to regulate endogenous signalling molecules affecting oncogenic pathways through glucuronidation, and possibly by alternative roles (see below) (Fig. 2b). The oncogenic state itself drastically perturbs the intratumoural expression of UGTs relative to normal tissues, as repeatedly reported based on mRNA, protein and/or enzymatic assays in untreated cancer patients.^17,29,31,33,50,53–69^ The expression of UGTs is repressed in certain tumour types relative to their normal tissue counterparts, but remarkably enhanced in other cancers, such as those of the prostate, pancreas, lung, endometrium and in CLL, indicating diverse patterns of regulation of UGT expression in tumours.^17,29,31,33,50,53–69^ These perturbed levels of UGTs are consistent with altered metabolic functions in tumours and suggest that UGTs might influence cancer progression, independent of exposure to therapeutic drugs (Table 1). Notably, in recent reports investigating metabolic perturbations present in the transcriptome and metabolome of multiple tumour types,^70,71^ the genes and metabolites for which levels are most perturbed belong to the pentose and glucuronate interconversion pathway that includes all UGT genes. These observations support the concept of a perturbed UGT pathway in several cancers.

**Table 1:** UGTs as prognostic biomarkers of cancer patient outcomes.

UGT	Approach^a^	Main observations	Refs
*Chronic lymphocytic leukaemia (CLL)*			
UGT2B17	RNA expression (*n* = 320)	Higher expression associated with shorter treatment-free survival and poor drug response	^50^
UGT2B17	RNA expression (*n* = 253)	Higher expression associated with shorter treatment-free and overall survival	^57^
*Prostate*			
UGT2B15	RNA expression (*n* = 55)	Higher expression in metastasis versus localised disease	^64^
UGT2B15 UGT2B17	RNA expression (*n* = 26)	Higher expression in metastasis versus localised disease	^62^
UGT2B15 UGT2B17	RNA and protein expression (*n* = 243)	Expression is not associated with Gleason score or disease stage	^63^
UGT2B28	Protein expression (*n* = 239)	Higher expression increases risk of recurrence after prostatectomy	^53^
UGT2B17 UGT2B28	DNA variations (*n* = 846)	Germline deletion increases risk of biochemical recurrence after prostatectomy	^76^
UGT2B15 UGT2B17	RNA expression (*n* = 179)	Higher expression in metastatic cases Negative correlation with prostate-specific antigen levels	^61^
UGT2B15 UGT2B17 UGT2B28	Protein expression (*n* = 190)	Higher expression linked to higher risk of biochemical recurrence after prostatectomy	^58^
UGT2B17	RNA and protein expression (*n* = 287)	Higher expression linked to higher Gleason score and risk of castration-resistant prostate cancer	^60^
UGT2B17	Protein expression (*n* = 239)	Higher expression linked to recurrence after prostatectomy and risk of metastasis	^59^
*Bladder*			
UGT1A	RNA and protein expression (*n* = 145)	Lower expression linked to recurrence/progression Inverse correlation with tumour grade/stage	^31^
*Breast*			
UGT2B15 UGT2B17	RNA expression (*n* = 2000)	Lower expression in higher tumour grade Expression associated with survival in distinct subgroups	^55^
UGT8	RNA expression (*n* = 744)	Higher expression linked to the risk of metastasis to the lung^b^	^68^
UGT8	RNA (*n* = 761) and protein (*n* = 40) expression	Higher expression linked to shorter lung-metastasis-free survival	^65^
*Head and neck*			
UGT2B17	DNA variations (*n* = 234)	Germline deletion combined with *TP53* mutations in primary tumours increases risk of relapse after surgery	^99^

^a^RNA expression was assessed by microarray or reverse transcription-quantitative PCR.

Protein expression was assessed by immunohistochemistry.

Germline DNA variations were assessed by PCR amplification of a genomic region encompassing the gene deletion.

^b^UGT8 expression was part of a six-gene signature predictive of metastasis.

The complex pattern of regulation of UGT expression (reviewed in ref. ^2^) involves the production of many alternative variant isoforms presenting novel in-frame sequences, which are differentially expressed in oncogenic states.^1,72^ Furthermore, clinical parameters, including the type of cancer, aggressiveness or stage and cell-type-specific expression, all contribute to this variability in UGT expression. These factors, coupled with the wide inter-individual variability and ethnic differences that also characterise variable UGT expression in tissues,^1^ suggest a multifaceted regulation of UGT expression by oncogenic conditions, which, in turn, will significantly influence not only the drug response, but also tumour progression, as described below.

Given that the activity of a number of UGTs determines exposure to endogenous hormone-like signalling molecules such as sex steroids under homoeostatic conditions (as outlined in the ‘Background' section), the best examples of the influence of UGTs on cancer progression arise from endocrine-related cancers. However, because UGTs conjugate GlcA to a host of other metabolites that trigger cancer-cell growth and death, including bile acids, retinoic acid and signalling lipids,^73^ one can envision that UGTs will influence cancers by regulating the local concentration of pro- and anti-oncogenic metabolites.

### UGTs in the progression of prostate cancer

**Fig. 4:**
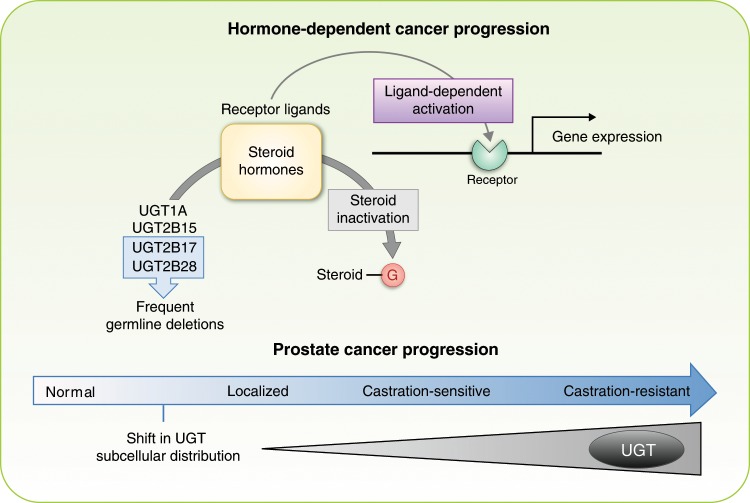
Cancer progression and UGTs: the example of prostate cancer. The glucuronidation pathway modulates the bioactivity of steroid hormones. The steroid-receptor-dependent activation of gene expression is in turn influenced by several UGTs whose activity is regulated by multiple factors, including germline deletions and transcriptional and translational regulation. Note that functions of UGTs unrelated to their glucuronidation activity discussed in the section ‘Metabolic influence of UGTs on cancer progression’ are not represented. In normal prostate tissues, UGT2B17 and UGT2B28 are predominantly nuclear enzymes, whereas in prostate tumour cells, nuclear and cytoplasmic distributions are observed.^53,98^ UGT expression is also reported to increase with prostate cancer progression and metastasis and influences patient outcome.^53,60,64,75,98^

UGT2B17 and UGT2B28 are two enzymes involved in the inactivation of steroids in target cells (along with UGT2B15), and are among the most commonly deleted genes of the human genome.^74^ There has been an ongoing interest in the association between their expression in hormone-related cancers and disease phenotypes, particularly in prostate cancer, given their ability to halt local steroid hormone signalling and the dependence of prostate cancer on androgens for proliferation and progression (Table 1; Fig. 4).^53,58,60,62,75–77^ A 2013 meta-analysis established that germline *UGT2B17* deletion (inherited deficiency) is associated with an increased risk of prostate cancer.^78^ Such data are lacking for the less common *UGT2B28* deletion polymorphism. Moreover, *UGT2B17* and *UGT2B28* germline deletions are both associated with biochemical recurrence after prostatectomy in men treated for newly diagnosed localised prostate cancer.^76^ These inherited deletions of *UGT2B17* and *UGT2B28* influence the circulating levels of sex steroids (androgens and oestrogens) in men with prostate cancer and are independent predictors of outcomes.^76^

The somatic loss of *UGT2B17* in tumour cells is associated with the development of a castration-resistant phenotype in vitro and in xenograft mouse models, consistent with the importance of this pathway in ligand-dependent pre-androgen receptor control of androgen metabolism.^79^ Indeed, inherited or acquired loss or inhibition of the UGT androgen inactivation pathway increases exposure to local androgens, enhances androgen receptor activation and promotes cancer-cell proliferation.^80^ However, there is accumulating evidence that UGT overexpression could function as an independent prognostic factor associated with the progression of prostate cancer.^53,60,64,75^ Microarray and proteomic analyses suggest that the expression levels of UGTs gradually increase from normal prostate tissue to primary tumours to metastases (Fig. 4).^64,75^ It is plausible that an increased androgen-inactivating UGT pathway renders prostate cancer cells much less sensitive to androgen depletion, facilitating a shift towards ligand-independent androgen receptor activation. In addition, UGT2B17 overexpression is associated with progression of castration-resistant prostate cancer mediated by a ligand-independent androgen receptor activation mechanism involving the proto-oncogene tyrosine-protein kinase Src (c-*Src)* oncogene.^60^ One potential mechanism raised by the authors and distinct from its role in androgen inactivation, relates to the UGT2B17 protein interacting and activating c-Src, triggering downstream signal pathways.^60^ A study of patients with endometrial cancer, another endocrine-related cancer, reveals that UGT2B17 overexpression might downregulate levels of the pro-apoptotic Mcl-1 protein, and therefore, promote cancer-cell survival, also implying a potential role of UGTs independent of their catalytic actions.^54^

For UGT2B28, a study of 239 prostate cancer specimens reveals that UGT2B28 overexpression is associated with increased circulating levels of testosterone and with advanced tumour characteristics such as the Gleason score, tumour size and nodal status.^53^ Beyond these associations with prognostic factors, overexpression of UGT2B28 is an adverse and independent prognostic factor, with an increased risk of prostate cancer recurrence and/or death by nearly threefold, after adjustments for known prognostic markers.^53^ This study also reveals that the localisation of UGT2B28 changes from predominantly nuclear in normal cells to nuclear, perinuclear and cytoplasmic in cancer cells, which might be secondary to alternative splicing processes, post-translational modifications and/or interactions with unknown binding partners.^53,81–83^ This alternative localisation may be representative of multifunctional proteins (moonlighting proteins). This suggests that UGT2B28 may have additional roles in tumour cells and raises the question of whether steroid inactivation is responsible for this influence.^84^ Thus, contrary to our expectations, UGT2B overexpression might alter androgen dependency, favour an aggressive disease phenotype and also support prostate cancer progression.

### UGTs in the progression of CLL

The impact of UGT proteins on disease phenotypes and clinical outcomes might well extend beyond their recognised enzymatic functions and could potentially be isoform- and cancer-specific. For example, an initial study of 320 patients with CLL prior to therapy initiation showed that high *UGT2B17* mRNA levels in leukaemic B cells are strongly associated with shorter treatment-free and overall survival.^50^ A second report validated *UGT2B17* as an informative prognostic marker in a Scandinavian cohort of 253 CLL patients and also among CLL patients with a mutated immunoglobulin heavy-chain variable region gene, a group for which few prognostic indicators exist.^57^ These studies imply a relevant role of the UGT2B17 pathway in progressive CLL and provide novel prognostic information. Based on enzymatic assays performed on patient samples, transcriptional expression correlates with UGT2B17 catalytic activity, implying a possible link between poor CLL survival and enhanced UGT2B17 conjugation activity. However, this activity appears unrelated to the well-known function of UGT2B17 in regulating exposure to steroid hormones. Indeed, although we have uncovered a link between circulating steroid levels and survival in CLL patients, *UGT2B17* germline deletion or *UGT2B17* mRNA expression levels in leukaemic B cells from CLL patients did not affect this relationship.^85^ This suggests that UGT2B17 might exert pro-leukaemic effects either through the metabolism of other endogenous metabolites and/or independently of its enzymatic activity. We also evidenced that UGT2B17-dependent glucuronidation of prostaglandin E2 (PGE_2_) impairs anti-oncogenic PGE_2_ effects in leukaemic cells, thereby potentially contributing to disease progression in CLL patients with high levels of UGT2B17.^52^ Furthermore, given its predictive role in the drug response, as described above, UGT2B17 has a dual role in determining the fate of patients with CLL, given that it is linked to disease progression as well as drug resistance (discussed above).

## Metabolic influence of UGTs on cancer progression

By virtue of their UDP-glucuronosyltransferase activity, UGTs can alter the biological activity and mediate the elimination of many low-molecular-mass endogenous molecules. The characterisation of endogenous metabolites that are substrates of UGTs is not yet complete, and predictions of other substrates are lacking, particularly in the absence of three-dimensional structural information. In addition to cholesterol-derived molecules (androgens and oestrogens, as discussed above, and bile acids), several other signalling molecules are documented substrates for which glucuronidation would affect their bioavailability and bioactivity, with the potential to disrupt tumour biology. Vitamin A/retinoic acid, vitamin D, thyroid hormone and serotonin, as well as numerous signalling lipids (arachidonic acid, leukotriene B4, prostaglandins and eicosanoid precursors) are endogenous substrates with established oncogenic functions that might be altered by glucuronidation.^52,86–89^ For instance, the UGT2B17-dependent regulation of prostaglandin PGE_2_ bioactivity is suggested to influence leukaemic cell proliferation and migration, as described above.^52^

The glucuronidation activity of UGTs also has the potential to influence the cellular pools of UDP-sugars and pathways involved in their synthesis and usage. For instance, given that UDP-glucose, UDP-GlcA and UDP-xylose are derived from the glycolytic intermediate glucose 6-phosphate, UGT activity may disturb the energy metabolism on which cancer cells depend to grow efficiently. A further impact of UGT activity might also involve the differential use of UDP-GlcA either for glucuronidation or for the synthesis of UDP-xylose and proteoglycans. This hypothesis has been raised in the context of prostate cancer progression to metastasis. Observations in prostate cancer-cell models suggest that UDP-GlcA is preferentially channelled for the synthesis of proteoglycans such as NOTCH1 in androgen-independent cells, possibly to avoid inactivation of intracellular pools of androgens.^90^ Finally, UGT activity also appears to modulate the synthesis of the glycosaminoglycan hyaluronan (HA), a constituent of the extracellular matrix, composed of GlcA units. HA possesses structural and cell-signalling functions that can affect the metastatic process by facilitating cell–cell signalling and motility.^91^ Enhanced HA levels confer an unfavourable prognosis (aggressive phenotypes and reduced survival) in several cancer types (reviewed in ref. ^92^). The synthesis of HA is strongly inhibited by 4-methylumbelliferone, a derivative of coumarin and a ubiquitous UGT substrate. One mechanism by which 4-methylumbelliferone impairs HA synthesis is attributed to the extensive glucuronidation of 4-methylumbelliferone by several UGTs that depletes pools of UDP-GlcA, the source of the GlcA moieties in HA (reviewed in ref. ^93^).

Beyond the enzymatic functions of UGTs in glucuronidation, their interactions with other metabolic enzymes, such as those involved in the catabolism of fatty acids and with the glycolytic enzyme pyruvate kinase (PKM2), are another means by which they might influence diverse metabolic pathways involved in cancer biology, with an impact on cancer-cell phenotypes.^81,82^ These additional functions, which could also involve UGT isoforms produced by alternative splicing, will need to be assessed to comprehensively understand the contribution of UGTs to the oncogenic phenotype.

## Conclusions

Perturbed UGT expression undoubtedly has an important influence on the response of cells to endogenous and exogenous factors, influencing cancer risk and progression in common malignancies as well as in drug response. The genetic status of UGTs could be relevant to refine the choice of personalised therapy.^15,94,95^ A number of predictive genetic variants have been identified in the UGT pathway and could help to pharmacologically optimise drug treatment. For example, cancer patients genetically predisposed with decreased UGT1A1 activity are at higher risk for severe toxicity when treated with anti-cancer agents such as irinotecan used in metastatic colorectal cancer. For these patients, a reduction of the starting dose is recommended to increase treatment safety.^96^

Emerging preclinical and clinical evidence indicates that the UGT pathway differs between cancer and normal cells, with alteration in acute and chronic leukaemias, as well as in several solid tumours, affecting disease progression and patient outcomes. For instance, the UGT inactivation pathway of endogenous molecules such as steroids, may potentially serve as a useful marker to identify disease with aggressive potential. Alterations in UGT expression are also associated with primary and acquired resistance to anti-cancer drugs, suggesting that targeting this pathway may potentially enhance or restore drug response. However, the mechanisms of dysregulation and precise consequences of altered UGTs, their subcellular localisation and biological functions in cancer cells (which perhaps diverge from their transferase activity in some circumstances—‘moonlighting proteins’) and their effects on downstream oncogenic pathways remain to be carefully examined. Rather than UGTs having a general function in oncogenic processes, each UGT protein, including alternative isoforms, might have specific roles and multifunctional ability, given their regulated expression at the tissue and cellular level and their specific substrate preferences.

Further research is required to improve our understanding of the influence of this important metabolic pathway in cancer aggressiveness, progression and drug resistance. Addressing these gaps will be essential for adapting therapeutic action, the goal being to optimise drug responses and delay disease progression and relapse. Based on emerging and accumulating preclinical and clinical evidence discussed in this review, therapeutic approaches that target UGTs directly or indirectly might eventually prove useful in delaying progression, increasing the drug-related response, avoiding drug resistance and ultimately improving patient outcomes. Still, the development of selective UGT inhibitors is in its infancy, and has so far exploited molecules that could competitively inhibit the catalytic activity by interfering with the binding of the co-substrate UDP-GlcA. This knowledge gap greatly impedes the development of specific inhibitors needed to avoid interfering with other UDP-GlcA-dependent pathways, as those described above. The high-throughput screening of collections of small molecular fragments^97^ may provide alternative solutions for the selective inhibition of UGTs. Such inhibitors could be used in conjunction with anti-cancer drugs to reduce their inactivation by the UGT pathway and restore drug sensitivity.

## Supplementary information

Table S1

## Data Availability

Not applicable.
