# Supplementary material for: Emerging roles for UDP-glucuronosyltransferases in drug resistance and cancer progression
Source: Br J Cancer. 2020 Feb 12;122(9):1277–87. doi: 10.1038/s41416-019-0722-0 (PMC7188667; doi:10.1038/s41416-019-0722-0)
Supplement: Supplementary file 1 — Table S1 [file 41416_2019_722_MOESM1_ESM.docx]

**Supplementary Table S1: List of anti-cancer agents metabolised by glucuronidation**

| **Agent*** | **Drug class** | **Type of cancer^†^** | **UGT enzyme(s) involved^‡^** | **Ref.** |
| --- | --- | --- | --- | --- |
| Abiraterone | Hormonal | Prostate | 1A4 | ^1^ |
| Acalabrutinib | Targeted | CLL | 1A4 | ^2^ |
| Asciminib | Targeted | CLL, ALL | 1A3, 1A4, 2B7, 2B17 | ^3^ |
| Anastrozole | Hormonal | Breast | 1A4 | ^4^ |
| Axitinib | Targeted | Renal cell carcinoma | 1A1, 1A3, 1A4, 1A9 | ^5-7^ |
| Belinostat | Targeted | Peripheral T-cell lymphoma | 1A1, 2B7 | ^8^ |
| Bendamustin | Alkylating | Leukaemia, lymphoma | 1A4 | ^2^ |
| Bexarotene | Retinoid | Cutaneous T-cell lymphoma | nd | ^9^ |
| Bicalutamide | Hormonal | Prostate | 1A9 | ^10^ |
| Binimetinib | Targeted | Melanoma | 1A1 | ^11,12^ |
| Bleomycin | Antibiotic | Multiple types of cancer | nd | ^13^ |
| Camptothecin | Topo. inhibitor | Colorectal | 1A1, 1A6, 1A9 | ^14,15^ |
| Cerdulatinib | Targeted | Leukaemia, lymphoma | 1A4, 2B17 | ^2^ |
| Chlorambucil | Alkylating | Leukaemia, lymphoma | 1A4, 2B17 | ^2^ |
| Cobimetinib | Targeted | Melanoma | 2B7 | ^16^ |
| Cytarabine | Antimetabolites | Leukaemia, lymphoma | 1A | ^17^ |
| Dasatinib | Targeted | CLL, ALL | nd | ^18^ |
| Daunorubicin | Antibiotics | Leukaemia | nd | ^19^ |
| Doxorubicin | Antibiotics | Multiple types of cancer | nd | ^20,21^ |
| Duvelisib | Targeted | Leukaemia, lymphoma | 1A4 | ^2^ |
| Enasidenib | Targeted | AML | 1A1, 1A3, 1A4, 1A9, 2B7, 2B15 | ^22,23^ |
| Encorafenib | Targeted | Melanoma | 1A1 | ^11^ |
| Epirubicin | Antibiotics | Breast | 2B7 | ^24^ |
| Erlotinib | Targeted | NSCLC, pancreas | nd | ^25^ |
| Etoposide | Topo. inhibitor | Multiple types of cancer | 1A1 | ^26,27^ |
| Exemestane | Hormonal | Breast | 1A4, 1A8, 1A10, 2B17 | ^28^ |
| Fenretinide | Retinoid | Breast | 1A1, 1A3, 1A6 | ^29^ |
| Flavopiridol | Targeted | ALL | 1A1, 1A9, 1A10 | ^30^ |
| Fludarabine | Antimetabolites | CLL | 1A4, 2B17 | ^2^ |
| 5-Fluorouracil | Antimetabolites | Colorectal | nd | ^31^ |
| Fluoxymesterone | Hormonal | Breast | nd | ^32^ |
| Flumatinib | Targeted | CML | nd | ^33^ |
| Fostamatinib | Targeted | DLBCL | 1A9 | ^34^ |
| Fulvestrant | Hormonal | Breast | 1A1, 1A3, 1A4, 1A8 | ^35^ |
| Glasdegib | Targeted | AML | 1A9 | ^11^ |
| Ibrutinib | Targeted | Leukaemia | 1A4 | ^2,36^ |
| Idelalisib | Targeted | CLL | 1A4 | ^2,37^ |
| Imatinib | Targeted | CML | 1A4, 2B10 | ^38^ |
| Irinotecan | Topo. inhibitor | Colorectal | 1A1, 1A7, 1A9, 1A10 | ^39^ |
| Isotretinoin | Retinoid | Skin | nd | ^40^ |
| Larotrectinib | Targeted | Multiple types of advanced solid tumours with NTRK fusion | nd | ^41^ |
| Letrozole | Hormonal | Breast | 1A4, 1A9, 2B4, 2B7, 2B17 | ^42^ |
| Lorlatinib | Targeted | NSCLC | 1A3 | ^11^ |
| Medroxyprogesterone | Hormonal | Endometrium | nd | ^43^ |
| Megestrol | Hormonal | Breast, endometrium | Multiple UGTs | ^44^ |
| Methotrexate | Antimetabolites | Multiple types of cancer | 1A | ^31^ |
| Mitoxantrone | Antibiotics | Leukaemia, prostate | nd | ^45^ |
| Nintedanib | Targeted | NSCLC | 1A1, 1A7, 1A8, 1A10 | ^46,47^ |
| Niraparib | Targeted | Ovarian | nd | ^48^ |
| Olaparib | Targeted | Breast, ovarian | nd | ^49^ |
| Palbociclib | Targeted | Breast | nd | ^50^ |
| Panobinostat | Targeted | Multiple myeloma | Multiple UGTs | ^51^ |
| Pomalidomide | Antiangiogenic | Multiple myeloma | nd | ^52^ |
| Raloxifene | Hormonal | Breast | 1A1, 1A8, 1A9, 1A10 | ^53,54^ |
| Regorafenib | Targeted | Colorectal, gastrointestinal, HCC | 1A9 | ^55,56^ |
| Ribavirin | Antimetabolites | Leukaemia, lymphoma | 1A | ^17^ |
| Ruxolitinib | Targeted | Rare bone marrow cancer | nd | ^57^ |
| Selinexor | Targeted | Multiple myeloma | nd | ^58^ |
| Sorafenib | Targeted | Renal | 1A9, 2B7 | ^59^ |
| Sunitinib | Targeted | Renal | nd | ^31^ |
| Talazoparib | Targeted | Breast | nd | ^60^ |
| Tamoxifen | Hormonal | Breast | 1A4, 2B7, 2B15, 2B17 | ^61,62^ |
| Thalidomide | Antiangiogenic | Multiple myeloma | nd | ^63^ |
| Tipifarnib | Targeted | AML | nd | ^64,65^ |
| Topotecan | Topo. inhibitor | Ovarian, cervical, small cell lung | nd | ^66,67^ |
| Toremifene | Hormonal | Breast | 1A4 | ^68^ |
| Trabectedin | Alkylating | Soft-tissue sarcomas, ovarian | 1A1, 2B15 | ^69,70^ |
| Trametinib | Targeted | Melanoma, NSCLC, thyroid | nd | ^71,72^ |
| Tretinoin | Retinoid | AML | 2B7 | ^73^ |
| Vandetanib | Targeted | Medullary thyroid | nd | ^74,75^ |
| Venetoclax | Targeted | Leukaemia, lymphoma | 1A4 | ^2^ |
| Vismodegib | Targeted | Basal-cell carcinoma | nd | ^76,77^ |
| Vorinostat | Targeted | Cutaneous T cell lymphoma | 1A8, 1A9, 1A10, 2B17 | ^78,79^ |

*List of anticancer drugs is not exhaustive

†Main indication

‡UGTs may be involved in the direct metabolism of the drug and/or its metabolites. The order of listed UGTs does not reflect their relative importance in the conjugation.

ALL: Acute lymphocytic leukaemia; AML: Acute myeloid leukaemia; CLL: Chronic lymphocytic leukaemia; CML: Chronic myeloid leukaemia; DLBCL: Diffuse large B-cell lymphoma; HCC: Hepatocellular carcinoma; NSCLC: Non-small cell lung carcinoma; nd: not determined/not available; NTRK: neurotrophic receptor tyrosine kinase; Topo. inhibitor: topoisomerase inhibitor.

**References**

1. Abiraterone. https://[www.janssen.com/canada/sites/www_janssen_com_canada/files/prod_files/live/zytiga_cpm.pdf](http://www.janssen.com/canada/sites/www_janssen_com_canada/files/prod_files/live/zytiga_cpm.pdf).

2. Allain EP, Rouleau M, Vanura K, Vaillancourt J, Bat V, Caron P *et al.* UGT2B17 Modifies Drug Response in Chronic Lymphocytic Leukemia. 2019, In revision.

3. Tran P, Hanna I, Eggimann FK, Schoepfer J, Ray T, Zhu B *et al.* Disposition of asciminib, a potent BCR-ABL1 tyrosine kinase inhibitor, in healthy male subjects. Xenobiotica 2019, 1-20.

4. Kamdem LK, Liu Y, Stearns V, Kadlubar SA, Ramirez J, Jeter S *et al.* In vitro and in vivo oxidative metabolism and glucuronidation of anastrozole. Br J Clin Pharmacol 2010, 70, 854-869.

5. Axitinib. https://[www.pfizer.ca/sites/g/files/g10056681/f/201904/INLYTA_PM_E_DC_207338_13Mar2019.pdf](http://www.pfizer.ca/sites/g/files/g10056681/f/201904/INLYTA_PM_E_DC_207338_13Mar2019.pdf).

6. Chen Y, Tortorici MA, Garrett M, Hee B, Klamerus KJ, Pithavala YK. Clinical pharmacology of axitinib. Clin Pharmacokinet 2013, 52, 713-725.

7. Zientek MA, Goosen TC, Tseng E, Lin J, Bauman JN, Walker GS *et al.* In Vitro Kinetic Characterization of Axitinib Metabolism. Drug Metab Dispos 2016, 44, 102-114.

8. Belinostat. https://[www.accessdata.fda.gov/drugsatfda_docs/label/2014/206256lbl.pdf](http://www.accessdata.fda.gov/drugsatfda_docs/label/2014/206256lbl.pdf).

9. Howell SR, Shirley MA, Grese TA, Neel DA, Wells KE, Ulm EH. Bexarotene metabolism in rat, dog, and human, synthesis of oxidative metabolites, and in vitro activity at retinoid receptors. Drug Metab Dispos 2001, 29, 990-998.

10. Grosse L, Paquet S, Caron P, Fazli L, Rennie PS, Belanger A *et al.* Androgen glucuronidation: an unexpected target for androgen deprivation therapy, with prognosis and diagnostic implications. Cancer Res 2013, 73, 6963-6971.

11. Badowski ME, Burton B, Shaeer KM, Dicristofano J. Oral oncolytic and antiretroviral therapy administration: dose adjustments, drug interactions, and other considerations for clinical use. Drugs Context 2019, 8, 212550.

12. Koelblinger P, Dornbierer J, Dummer R. A review of binimetinib for the treatment of mutant cutaneous melanoma. Future Oncol 2017, 13, 1755-1766.

13. Kocan F, Avcibasi U, Unak P, Muftuler FZ, Ichedef CA, Demiroglu H *et al.* Metabolic comparison of radiolabeled bleomycin and bleomycin-glucuronide labeled with 99mTc. Cancer Biother Radiopharm 2011, 26, 573-584.

14. Cummings J, Boyd G, Ethell BT, Macpherson JS, Burchell B, Smyth JF *et al.* Enhanced clearance of topoisomerase I inhibitors from human colon cancer cells by glucuronidation. Biochem Pharmacol 2002, 63, 607-613.

15. Cummings J, Zelcer N, Allen JD, Yao D, Boyd G, Maliepaard M *et al.* Glucuronidation as a mechanism of intrinsic drug resistance in colon cancer cells: contribution of drug transport proteins. Biochem Pharmacol 2004, 67, 31-39.

16. Cobimetinib. https://pdf.hres.ca/dpd_pm/00043025.PDF.

17. Zahreddine HA, Culjkovic-Kraljacic B, Assouline S, Gendron P, Romeo AA, Morris SJ *et al.* The sonic hedgehog factor GLI1 imparts drug resistance through inducible glucuronidation. Nature 2014, 511, 90-93.

18. Christopher LJ, Cui D, Li W, Barros A, Jr., Arora VK, Zhang H *et al.* Biotransformation of [14C]dasatinib: in vitro studies in rat, monkey, and human and disposition after administration to rats and monkeys. Drug Metab Dispos 2008, 36, 1341-1356.

19. Gessner T, Vaughan LA, Beehler BC, Bartels CJ, Baker RM. Elevated pentose cycle and glucuronyltransferase in daunorubicin-resistant P388 cells. Cancer Res 1990, 50, 3921-3927.

20. Lal D, Park JA, Demock K, Marinaro J, Perez AM, Lin MH *et al.* Aflibercept exerts antivascular effects and enhances levels of anthracycline chemotherapy in vivo in human acute myeloid leukemia models. Mol Cancer Ther 2010, 9, 2737-2751.

21. Weenen H, van Maanen JM, de Planque MM, McVie JG, Pinedo HM. Metabolism of 4'-modified analogs of doxorubicin. unique glucuronidation pathway for 4'-epidoxorubicin. Eur J Cancer Clin Oncol 1984, 20, 919-926.

22. Enasidenib. https://pdf.hres.ca/dpd_pm/00049562.PDF.

23. Tong Z, Atsriku C, Yerramilli U, Wang X, Li Y, Reyes J *et al.* Absorption, distribution, metabolism and excretion of an isocitrate dehydrogenase-2 inhibitor enasidenib in rats and humans. Xenobiotica 2019, 49, 200-210.

24. Innocenti F, Schilsky RL, Ramirez J, Janisch L, Undevia S, House LK *et al.* Dose-finding and pharmacokinetic study to optimize the dosing of irinotecan according to the UGT1A1 genotype of patients with cancer. J Clin Oncol 2014, 32, 2328-2334.

25. Ling J, Johnson KA, Miao Z, Rakhit A, Pantze MP, Hamilton M *et al.* Metabolism and excretion of erlotinib, a small molecule inhibitor of epidermal growth factor receptor tyrosine kinase, in healthy male volunteers. Drug Metab Dispos 2006, 34, 420-426.

26. Etoposide. https://pdf.hres.ca/dpd_pm/00038429.PDF.

27. Wen Z, Tallman MN, Ali SY, Smith PC. UDP-glucuronosyltransferase 1A1 is the principal enzyme responsible for etoposide glucuronidation in human liver and intestinal microsomes: structural characterization of phenolic and alcoholic glucuronides of etoposide and estimation of enzyme kinetics. Drug Metab Dispos 2007, 35, 371-380.

28. Lazarus P, Sun D. Potential role of UGT pharmacogenetics in cancer treatment and prevention: focus on tamoxifen and aromatase inhibitors. Drug Metab Rev 2010, 42, 182-194.

29. Illingworth NA, Boddy AV, Daly AK, Veal GJ. Characterization of the metabolism of fenretinide by human liver microsomes, cytochrome P450 enzymes and UDP-glucuronosyltransferases. Br J Pharmacol 2011, 162, 989-999.

30. Hagenauer B, Salamon A, Thalhammer T, Kunert O, Haslinger E, Klingler P *et al.* In vitro glucuronidation of the cyclin-dependent kinase inhibitor flavopiridol by rat and human liver microsomes: involvement of UDP-glucuronosyltransferases 1A1 and 1A9. Drug Metab Dispos 2001, 29, 407-414.

31. Zahreddine HA, Culjkovic-Kraljacic B, Gasiorek J, Duchaine J, Borden KLB. GLI1-Inducible Glucuronidation Targets a Broad Spectrum of Drugs. ACS Chem Biol 2019, 14, 348-355.

32. Fluoxymesterone. https://dailymed.nlm.nih.gov/dailymed/drugInfo.cfm?setid=5328c194-5650-4d1f-9e28-8cea038cce81.

33. Gong A, Chen X, Deng P, Zhong D. Metabolism of flumatinib, a novel antineoplastic tyrosine kinase inhibitor, in chronic myelogenous leukemia patients. Drug Metab Dispos 2010, 38, 1328-1340.

34. Clemons Bankston P, Al-Horani RA. New Small Molecule Drugs for Thrombocytopenia: Chemical, Pharmacological, and Therapeutic Use Considerations. Int J Mol Sci 2019, 20.

35. Fulvestrant. https://[www.astrazeneca.ca/content/dam/az-ca/downloads/productinformation/faslodex-product-monograph-en.pdf](http://www.astrazeneca.ca/content/dam/az-ca/downloads/productinformation/faslodex-product-monograph-en.pdf).

36. Scheers E, Leclercq L, de Jong J, Bode N, Bockx M, Laenen A *et al.* Absorption, metabolism, and excretion of oral (1)(4)C radiolabeled ibrutinib: an open-label, phase I, single-dose study in healthy men. Drug Metab Dispos 2015, 43, 289-297.

37. Ramanathan S, Jin F, Sharma S, Kearney BP. Clinical Pharmacokinetic and Pharmacodynamic Profile of Idelalisib. Clin Pharmacokinet 2016, 55, 33-45.

38. Lu D, Xie Q, Wu B. N-glucuronidation catalyzed by UGT1A4 and UGT2B10 in human liver microsomes: Assay optimization and substrate identification. J Pharm Biomed Anal 2017, 145, 692-703.

39. Irinotecan. https://[www.pfizer.ca/sites/g/files/g10050796/f/201903/Irinotecan_HCL_USP_PM_E_224931_08Mar2019.pdf](http://www.pfizer.ca/sites/g/files/g10050796/f/201903/Irinotecan_HCL_USP_PM_E_224931_08Mar2019.pdf).

40. Sonawane P, Cho HE, Tagde A, Verlekar D, Yu AL, Reynolds CP *et al.* Metabolic characteristics of 13-cis-retinoic acid (isotretinoin) and anti-tumour activity of the 13-cis-retinoic acid metabolite 4-oxo-13-cis-retinoic acid in neuroblastoma. Br J Pharmacol 2014, 171, 5330-5344.

41. Scott LJ. Larotrectinib: First Global Approval. Drugs 2019, 79, 201-206.

42. Precht JC, Schroth W, Klein K, Brauch H, Krynetskiy E, Schwab M *et al.* The letrozole phase 1 metabolite carbinol as a novel probe drug for UGT2B7. Drug Metab Dispos 2013, 41, 1906-1913.

43. Medroxyprogesterone. https://[www.pfizer.ca/sites/g/files/g10050796/f/201710/Provera_PM_E_176877_29September2014.pdf](http://www.pfizer.ca/sites/g/files/g10050796/f/201710/Provera_PM_E_176877_29September2014.pdf)

44. House L, Seminerio MJ, Mirkov S, Ramirez J, Skor M, Sachleben JR *et al.* Metabolism of megestrol acetate in vitro and the role of oxidative metabolites. Xenobiotica 2018, 48, 973-983.

45. Mitoxanthrone. https://[www.fresenius-kabi.com/en-ca/documents/Mitoxantrone-PM-Eng-v2.1-clean.pdf](http://www.fresenius-kabi.com/en-ca/documents/Mitoxantrone-PM-Eng-v2.1-clean.pdf).

46. Nintedanib. https://[www.boehringer-ingelheim.ca/sites/ca/files/documents/ofevpmen.pdf](http://www.boehringer-ingelheim.ca/sites/ca/files/documents/ofevpmen.pdf)

47. Stopfer P, Rathgen K, Bischoff D, Ludtke S, Marzin K, Kaiser R *et al.* Pharmacokinetics and metabolism of BIBF 1120 after oral dosing to healthy male volunteers. Xenobiotica 2011, 41, 297-311.

48. Niraparib. https://[www.zejula.com/prescribing-information](http://www.zejula.com/prescribing-information).

49. Olaparib. https://[www.astrazeneca.ca/content/dam/az-ca/downloads/productinformation/lynparza-tablet-product-monograph-en.pdf](http://www.astrazeneca.ca/content/dam/az-ca/downloads/productinformation/lynparza-tablet-product-monograph-en.pdf).

50. Palbociclib. https://[www.pfizer.ca/sites/g/files/g10050796/f/201806/IBRANCE_PM_207909_5June2018_E.pdf](http://www.pfizer.ca/sites/g/files/g10050796/f/201806/IBRANCE_PM_207909_5June2018_E.pdf).

51. Panobinostat. https://[www.pharma.us.novartis.com/sites/www.pharma.us.novartis.com/files/farydak.pdf](http://www.pharma.us.novartis.com/sites/www.pharma.us.novartis.com/files/farydak.pdf).

52. Shimizu M, Suemizu H, Mitsui M, Shibata N, Guengerich FP, Yamazaki H. Metabolic profiles of pomalidomide in human plasma simulated with pharmacokinetic data in control and humanized-liver mice. Xenobiotica 2017, 47, 844-848.

53. Kokawa Y, Kishi N, Jinno H, Tanaka-Kagawa T, Narimatsu S, Hanioka N. Effect of UDP-glucuronosyltransferase 1A8 polymorphism on raloxifene glucuronidation. Eur J Pharm Sci 2013, 49, 199-205.

54. Sun D, Jones NR, Manni A, Lazarus P. Characterization of raloxifene glucuronidation: potential role of UGT1A8 genotype on raloxifene metabolism in vivo. Cancer Prev Res (Phila) 2013, 6, 719-730.

55. Gerisch M, Hafner FT, Lang D, Radtke M, Diefenbach K, Cleton A *et al.* Mass balance, metabolic disposition, and pharmacokinetics of a single oral dose of regorafenib in healthy human subjects. Cancer Chemother Pharmacol 2018, 81, 195-206.

56. Regorafenib. https://[www.bayer.ca/omr/online/stivarga-pm-en.pdf](http://www.bayer.ca/omr/online/stivarga-pm-en.pdf).

57. Shilling AD, Nedza FM, Emm T, Diamond S, McKeever E, Punwani N *et al.* Metabolism, excretion, and pharmacokinetics of [14C]INCB018424, a selective Janus tyrosine kinase 1/2 inhibitor, in humans. Drug Metab Dispos 2010, 38, 2023-2031.

58. Abdul Razak AR, Mau-Soerensen M, Gabrail NY, Gerecitano JF, Shields AF, Unger TJ *et al.* First-in-Class, First-in-Human Phase I Study of Selinexor, a Selective Inhibitor of Nuclear Export, in Patients With Advanced Solid Tumors. J Clin Oncol 2016, 34, 4142-4150.

59. Ye L, Yang X, Guo E, Chen W, Lu L, Wang Y *et al.* Sorafenib metabolism is significantly altered in the liver tumor tissue of hepatocellular carcinoma patient. PLoS One 2014, 9, e96664.

60. Talazoparib. <http://labeling.pfizer.com/ShowLabeling.aspx?id=11046#section-11.2>.

61. Romero-Lorca A, Novillo A, Gaibar M, Bandres F, Fernandez-Santander A. Impacts of the Glucuronidase Genotypes UGT1A4, UGT2B7, UGT2B15 and UGT2B17 on Tamoxifen Metabolism in Breast Cancer Patients. PLoS One 2015, 10, e0132269.

62. Sutiman N, Lim JSL, Muerdter TE, Singh O, Cheung YB, Ng RCH *et al.* Pharmacogenetics of UGT1A4, UGT2B7 and UGT2B15 and Their Influence on Tamoxifen Disposition in Asian Breast Cancer Patients. Clin Pharmacokinet 2016, 55, 1239-1250.

63. Lu J, Helsby N, Palmer BD, Tingle M, Baguley BC, Kestell P *et al.* Metabolism of thalidomide in liver microsomes of mice, rabbits, and humans. J Pharmacol Exp Ther 2004, 310, 571-577.

64. Garner RC, Goris I, Laenen AA, Vanhoutte E, Meuldermans W, Gregory S *et al.* Evaluation of accelerator mass spectrometry in a human mass balance and pharmacokinetic study-experience with 14C-labeled (R)-6-[amino(4- chlorophenyl)(1-methyl-1H-imidazol-5-yl)methyl]-4-(3-chlorophenyl)-1- methyl-2(1H)-quinolinone (R115777), a farnesyl transferase inhibitor. Drug Metab Dispos 2002, 30, 823-830.

65. Zhang S, Zannikos P, Awada A, Piccart-Gebhart M, Dirix LY, Fumoleau P *et al.* Pharmacokinetics of tipifarnib after oral and intravenous administration in subjects with advanced cancer. J Clin Pharmacol 2006, 46, 1116-1127.

66. Rosing H, van Zomeren DM, Doyle E, Bult A, Beijnen JH. O-glucuronidation, a newly identified metabolic pathway for topotecan and N-desmethyl topotecan. Anticancer Drugs 1998, 9, 587-592.

67. Topotecan. https://[www.sandoz.ca/sites/www.sandoz.ca/files/Topotecan%20Product%20Monograph.pdf](http://www.sandoz.ca/sites/www.sandoz.ca/files/Topotecan%20Product%20Monograph.pdf).

68. Mazzarino M, de la Torre X, Botre F. Urinary excretion profiles of toremifene metabolites by liquid chromatography-mass spectrometry. Towards targeted analysis to relevant metabolites in doping control. Anal Bioanal Chem 2011, 401, 529-541.

69. Beumer JH, Rademaker-Lakhai JM, Rosing H, Hillebrand MJ, Bosch TM, Lopez-Lazaro L *et al.* Metabolism of trabectedin (ET-743, Yondelis) in patients with advanced cancer. Cancer Chemother Pharmacol 2007, 59, 825-837.

70. Trabectedin. https://[www.janssen.com/canada/sites/www_janssen_com_canada/files/prod_files/live/yondelis_cpm.pdf](http://www.janssen.com/canada/sites/www_janssen_com_canada/files/prod_files/live/yondelis_cpm.pdf)

71. Ho MY, Morris MJ, Pirhalla JL, Bauman JW, Pendry CB, Orford KW *et al.* Trametinib, a first-in-class oral MEK inhibitor mass balance study with limited enrollment of two male subjects with advanced cancers. Xenobiotica 2014, 44, 352-368.

72. Trametinib. https://[www.novartis.ca/sites/www.novartis.ca/files/mekinist_scrip_e.pdf](http://www.novartis.ca/sites/www.novartis.ca/files/mekinist_scrip_e.pdf).

73. Gestl SA, Green MD, Shearer DA, Frauenhoffer E, Tephly TR, Weisz J. Expression of UGT2B7, a UDP-glucuronosyltransferase implicated in the metabolism of 4-hydroxyestrone and all-trans retinoic acid, in normal human breast parenchyma and in invasive and in situ breast cancers. Am J Pathol 2002, 160, 1467-1479.

74. Martin P, Oliver S, Kennedy SJ, Partridge E, Hutchison M, Clarke D *et al.* Pharmacokinetics of vandetanib: three phase I studies in healthy subjects. Clin Ther 2012, 34, 221-237.

75. Vandetanib. <http://products.sanofi.ca/en/caprelsa-en.pdf>.

76. Khojasteh SC, Yue Q, Ma S, Castanedo G, Chen JZ, Lyssikatos J *et al.* Investigations into the mechanisms of pyridine ring cleavage in vismodegib. Drug Metab Dispos 2014, 42, 343-351.

77. Vismodegib. https://[www.rochecanada.com/PMs/Erivedge/Erivedge_PM_E.pdf](http://www.rochecanada.com/PMs/Erivedge/Erivedge_PM_E.pdf)

78. Balliet RM, Chen G, Gallagher CJ, Dellinger RW, Sun D, Lazarus P. Characterization of UGTs active against SAHA and association between SAHA glucuronidation activity phenotype with UGT genotype. Cancer Res 2009, 69, 2981-2989.

79. Kang SP, Ramirez J, House L, Zhang W, Mirkov S, Liu W *et al.* A pharmacogenetic study of vorinostat glucuronidation. Pharmacogenet Genomics 2010, 20, 638-641.
